# The Role of AI in Hospitals and Clinics: Transforming Healthcare in the 21st Century

**DOI:** 10.3390/bioengineering11040337

**Published:** 2024-03-29

**Authors:** Shiva Maleki Varnosfaderani, Mohamad Forouzanfar

**Affiliations:** 1 Department of Electrical Engineering, Wayne State University, Detroit, MI 48202, USA; 2 Département de Génie des Systèmes, École de Technologie Supérieure (ÉTS), Université du Québec, Montréal, QC H3C 1K3, Canada; 3 Centre de Recherche de L’institut Universitaire de Gériatrie de Montréal (CRIUGM), Montréal, QC H3W 1W5, Canada

**Keywords:** artificial intelligence, clinical decision-making, ethical considerations in healthcare AI, healthcare technology, medical imaging and diagnostics

## Abstract

As healthcare systems around the world face challenges such as escalating costs, limited access, and growing demand for personalized care, artificial intelligence (AI) is emerging as a key force for transformation. This review is motivated by the urgent need to harness AI’s potential to mitigate these issues and aims to critically assess AI’s integration in different healthcare domains. We explore how AI empowers clinical decision-making, optimizes hospital operation and management, refines medical image analysis, and revolutionizes patient care and monitoring through AI-powered wearables. Through several case studies, we review how AI has transformed specific healthcare domains and discuss the remaining challenges and possible solutions. Additionally, we will discuss methodologies for assessing AI healthcare solutions, ethical challenges of AI deployment, and the importance of data privacy and bias mitigation for responsible technology use. By presenting a critical assessment of AI’s transformative potential, this review equips researchers with a deeper understanding of AI’s current and future impact on healthcare. It encourages an interdisciplinary dialogue between researchers, clinicians, and technologists to navigate the complexities of AI implementation, fostering the development of AI-driven solutions that prioritize ethical standards, equity, and a patient-centered approach.

## 1. Introduction

In recent years, artificial intelligence (AI) has emerged as a transformative force in various sectors, with healthcare being one of the most significant [1]. The integration of AI into hospitals and clinics represents a paradigm shift in how medical care is delivered and managed. This paper aims to explore the multifaceted role of AI in healthcare settings, focusing on its impact on clinical decision-making, hospital operations, medical diagnostics, patient care, and the ethical considerations it raises.

The concept of AI in healthcare is not new; it dates back to the early days of computer science when researchers first envisioned machines capable of mimicking human intelligence [2]. However, it was not until the advancement of machine learning algorithms [3] and the exponential increase in computational power and data availability [4] that AI applications in healthcare truly began to flourish. This evolution has been marked by significant milestones, from early expert systems [5] to advanced neural networks capable of outperforming human experts in specific tasks [6].

Today, AI in healthcare encompasses a broad range of applications [7]. In clinical settings, it assists in diagnosing diseases, predicting patient outcomes, and personalizing treatment plans [8]. In hospital management, AI optimizes operational efficiency, streamlines administrative tasks, and improves patient flow and scheduling [9]. In the field of medical diagnostics, AI enhances the accuracy and speed of image analysis in radiology and pathology [10]. Moreover, AI plays a crucial role in patient care through remote monitoring, telemedicine, and virtual assistance, fundamentally altering the patient–doctor interaction paradigm [11].

**Figure 1:**
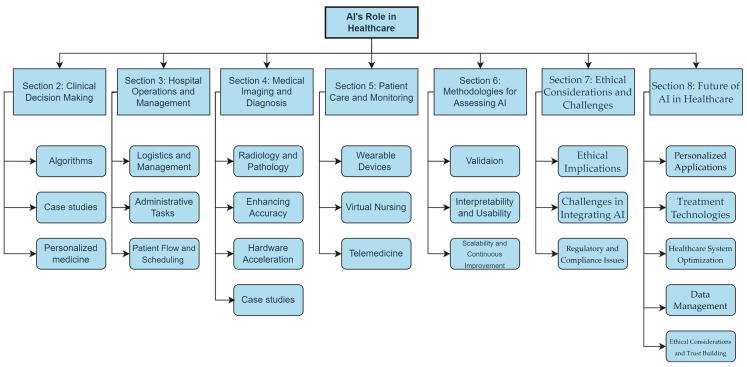
Comprehensive overview of AI applications in hospitals and clinics: detailed exploration of key topics addressed in this paper.

This paper explores artificial intelligence’s evolving role in healthcare, focusing on its application in hospitals and clinics. In consideration of the extensive scope of this study, we employed a meticulous approach in selecting references, focusing primarily on works published within reputable journals over the past five years. Our search was conducted using both Google Scholar and PubMed, ensuring a comprehensive exploration of the pertinent literature. Figure 1 provides a comprehensive overview of the key topics addressed in this paper. We start with AI in clinical decision-making, highlighting its use in diagnosis, prognosis, and personalized medicine through specific disease case studies. The discussion then moves to AI’s role in improving hospital operations and management, including logistics, administrative tasks, and scheduling. Further, we examine AI in medical imaging and diagnostics, where it enhances accuracy and efficiency in radiology and pathology. This paper also covers AI’s impact on patient care and monitoring, with a look at AI-powered wearables and virtual nursing assistants, and the expansion of telemedicine. We also discuss methodologies to assess the performance of AI healthcare solutions. Ethical considerations and challenges of AI integration, such as privacy, bias, and data security, are addressed, followed by a look at the future of AI in healthcare, considering its potential to improve patient outcomes and respond to global health crises.

## 2. AI in Clinical Decision-Making

This section explores how AI, with its advanced learning and processing capabilities, is reshaping the domain of medical diagnostics and treatment. By harnessing the power of AI, healthcare professionals are now equipped with tools that provide deeper insights into patient data. This leads to more accurate diagnoses and effective treatment plans. We will explore three critical aspects: AI algorithms for diagnosis and prognosis, case studies of AI in detecting diseases like cancer and diabetes, and AI’s role in the growing field of personalized medicine.

### 2.1. AI Algorithms for Diagnosis and Prognosis

AI algorithms are becoming important contributors in diagnosing and predicting diseases and offer new insights to healthcare. These algorithms analyze vast amounts of medical data to identify patterns and correlations that might elude human analysis. For instance, in oncology, AI algorithms can sift through radiographic images, genetic information, and patient histories to detect cancer at early stages [12]. Similarly, in the field of cardiology, AI models are employed to predict heart attacks and strokes by analyzing ECG patterns and other vital signs [13].

One of the key strengths of AI in diagnosis is its ability to continually learn and improve. As these algorithms are exposed to more data, their diagnostic precision and predictive accuracy are enhanced. This is crucial in managing complex and chronic diseases where early detection and timely intervention can be life-saving [14].

Moreover, AI’s role in prognosis is equally transformative [15,16]. By analyzing patterns in disease progression, AI can forecast potential complications, enabling healthcare professionals to devise preemptive strategies. This is particularly important in chronic diseases like diabetes, where AI can predict potential risks, such as kidney failure or vision loss, by analyzing blood sugar levels, lifestyle factors, and treatment responses over time [17].

AI algorithms can be broadly categorized into machine learning, deep learning, and natural language processing, each with unique strengths and applications:

1. Machine learning (ML): ML algorithms learn from data to make predictions or decisions without being explicitly programmed for the task [18]. In healthcare, supervised learning algorithms have been instrumental in developing predictive models for patient outcomes based on historical data [19]. Unsupervised learning, on the other hand, is used to identify patterns or clusters within data, useful in discovering novel disease subtypes [20]. Reinforcement learning, where algorithms learn to make sequences of decisions by trial and error, has potential in personalized treatment optimization [21].
2. Deep learning (DL): A subset of ML, deep learning uses neural networks with multiple layers (hence “deep”) to analyze complex data structures. Convolutional Neural Networks (CNNs) are particularly effective in processing imaging data, making them invaluable for diagnosing diseases from medical images like X-rays or MRIs [22]. Some other advanced CNN architectures include Residual Network (ResNet), Inception, Visual Geometry Group (VGG), and Graph Convolutional Networks (GCNs), each with its own strengths and applications in image analysis, classification, and graph data processing [23]. Recurrent Neural Networks (RNNs), known for their ability to handle sequential data, are used for analyzing time-series data, such as physiological signals collected during patient monitoring, to predict health deteriorations or outcomes over time [24]. For instance, Long Short-Term Memory (LSTM) networks, a sophisticated variant of RNNs, have been extensively utilized in the detection of sleep apnea using polysomnography data [25]. Additionally, Transformer models, such as BERT (Bidirectional Encoder Representations from Transformers) and GPT (Generative Pre-trained Transformer), offer revolutionary approaches to processing natural language in clinical notes, enabling more accurate extraction of patient information and insights. Generative Adversarial Networks (GANs) [26] and conditional diffusion models [27] have emerged as a powerful tool for generating synthetic medical images for training without privacy concerns, while Graph Neural Networks (GNNs) are unlocking new possibilities in modeling complex biological and health-related networks, from predicting protein interactions to understanding disease pathways.

Table 1 provides a summary of the various deep learning models discussed, including their applications, strengths, and areas of healthcare they are transforming.

3. Natural language processing (NLP): NLP algorithms allow computers to understand and interpret human language. In healthcare, NLP is used to extract meaningful information from unstructured data sources like clinical notes or the research literature, aiding in both diagnostic processes and the aggregation of knowledge for prognosis estimation [33]. An example of such a language model is the GatorTron [34]. It is a large-scale Transformer-based NLP model tailored for the healthcare domain. It utilizes the Transformer architecture, known for its efficiency in handling sequence-to-sequence tasks and its ability to process large datasets, to interpret and analyze electronic health records. With its 8.9 billion parameters, GatorTron is trained on over 90 billion words of clinical text, making it a highly advanced model for extracting and understanding complex medical information from unstructured data sources.

AI algorithms are not just tools for efficient diagnosis and prognosis; they represent a paradigm shift in understanding and managing health and disease. The next sections will provide deeper insights into specific case studies and the role of AI in personalizing medical care, further highlighting AI’s profound impact on clinical decision-making.

### 2.2. Case Studies of AI in Detecting Diseases

The potential of AI in the early detection and accurate diagnosis of diseases such as cancer, diabetes, and other critical conditions has been demonstrated in various case studies. This subsection explores some notable examples, illustrating how AI technology is making strides in the field of disease detection:

1. Cancer detection: One of the most groundbreaking applications of AI is in the early detection of cancer. A notable case study involves the use of deep learning algorithms in the analysis of mammograms for breast cancer detection. Research has shown that AI can identify patterns in mammographic images that are indicative of cancerous growths, often with greater accuracy than traditional methods. A notable study published in the journal Nature reported the development of an AI model by Google Health [35]. This model was trained on a large dataset of mammograms and demonstrated the ability to detect breast cancer more accurately than human radiologists. The AI system showed a reduction in both false positives and false negatives, key factors in cancer diagnostics. This progress in AI technology is significant because early detection of breast cancer can dramatically improve prognosis and treatment outcomes.
2. Diabetes management: AI’s role in managing and detecting diabetes, particularly through machine learning algorithms, is a significant area of improvement in healthcare. These algorithms can analyze patient data to predict the onset and progression of diabetes and its complications, as demonstrated in different studies.
  In one study, several supervised classification algorithms were applied to predict and classify eight diabetes complications, including metabolic syndrome, dyslipidemia, neuropathy, nephropathy, diabetic foot, hypertension, obesity, and retinopathy [36]. The dataset utilized in this study comprises 79 input attributes, including results of medical tests and demographic information collected from 884 patients. The performance of the models was evaluated using the accuracy and F1 score, reaching a maximum of 97.8% and 97.7%, respectively. Among different classifiers, random forest (RF), Adaboost, and XGBoost achieved the best performance. This high level of accuracy demonstrates the potential of machine learning in effectively predicting diabetes complications.
  Another study focused on evaluating the efficacy of machine learning algorithms in predicting complications and poor glycemic control in nonadherent type 2 diabetes patients [37]. This real-world study used data from 800 type 2 diabetes patients, of which 165 met the inclusion criteria. Different machine learning algorithms were used to develop prediction models, with the predictive performance assessed using the area under the curve. The highest performance scores for predicting various complications such as diabetic nephropathy, neuropathy, angiopathy, and eye disease were 90.2%, 85.9%, 88.9%, and 83.2%, showcasing the effectiveness of these models.
3. Heart disease prediction: The prediction of heart disease using AI represents a significant advancement in cardiovascular healthcare. This application not only aims to predict the occurrence of heart disease but also attempts to determine its severity, a crucial factor in effective treatment and management.
  One innovative study in this area focused on a machine learning-based prediction model that performs both binary and multiple classifications of heart disease [38]. The model, known as Fuzzy-GBDT, integrates fuzzy logic with a gradient boosting decision tree to streamline data complexity and improve prediction accuracy. Additionally, to avoid overfitting, the model incorporates a bagging technique, enhancing its capability to classify the severity of heart disease. The evaluation results of this model show excellent accuracy and stability in predicting heart disease, demonstrating its potential as a valuable tool in healthcare.
  Another interesting study introduces a cutting-edge healthcare system that employs ensemble deep learning coupled with feature fusion approaches [39]. This system is designed to overcome the limitations of traditional machine learning models that struggle with high-dimensional datasets. It achieves this by integrating sensor data with electronic medical records, creating a more holistic dataset for heart disease prediction. The system uses the information gain technique to streamline this dataset, focusing on the most relevant features and thereby reducing computational complexity. A key aspect of this model is the application of conditional probability for precise feature weighting, enhancing the overall performance of the system. Impressively, this ensemble deep learning model achieved an accuracy of 98.5%, outperforming existing models and illustrating its efficacy in heart disease prediction.
4. AI in neurological disorders: The integration of AI, particularly deep learning (DL), in neurology has opened new avenues for the diagnosis and management of neurological disorders. The recent literature reveals significant progress in employing AI for the early detection and more accurate diagnosis of various conditions, including AD.
  One area of notable advancement is the use of deep learning in neuroimaging studies. DL’s ability to process and learn from raw data through complex, nonlinear transformations makes it well suited for identifying the subtle and diffuse alterations characteristic of many neurological and psychiatric disorders. Research in this domain has shown that DL can be a powerful tool in the ongoing search for biomarkers of such conditions, offering potential breakthroughs in understanding and diagnosing brain-based disorders [40].
  Furthering this progress, a comprehensive review of deep learning techniques in the prognosis of a range of neuropsychiatric and neurological disorders, such as stroke, Alzheimer’s, Parkinson’s, epilepsy, autism, migraine, cerebral palsy, and multiple sclerosis, has underscored deep learning’s versatility in addressing real-life challenges across various domains, including disease diagnosis [41]. In the specific case of Alzheimer’s Disease (AD), the most common cause of dementia, deep learning has shown promise in enhancing diagnosis accuracy. Utilizing Convolutional Neural Networks (CNNs), researchers have developed frameworks for detecting AD characteristics from Magnetic Resonance Imaging (MRI) data [42]. By considering different stages of dementia and creating high-resolution disease probability maps, these models provide intuitive visualizations of individual AD risk. This approach, especially when addressing class imbalance in datasets, has achieved high accuracy, surpassing existing methods. The adaptation of such models to extensive datasets like the Alzheimer’s Disease Neuroimaging Initiative (ADNI) further validates their effectiveness in predicting AD classes.
5. Key insights: These case studies highlight AI’s significant role in advancing disease detection across multiple medical disciplines, offering accurate and timely diagnoses, often through non-invasive methods. However, as AI technology continues to evolve, there is a critical need for addressing challenges such as data privacy, algorithmic transparency, and ensuring equitable access to these technologies. Future developments should focus on creating more robust AI systems that can handle diverse datasets, thereby reducing potential biases in diagnosis. Additionally, integrating AI with traditional diagnostic methods and enhancing interdisciplinary collaboration among technologists, clinicians, and patients will be key to harnessing AI’s full potential in disease detection and management.

### 2.3. The Role of AI in Personalized Medicine

The advent of AI in healthcare has boosted the growth of personalized medicine, a paradigm that tailors medical treatment to the individual characteristics of each patient. This subsection explores how AI is instrumental in driving this personalized approach, offering new insights into patient care that were previously unattainable:

1. Tailoring treatments to genetic profiles: One of the most significant applications of AI in personalized medicine is in the field of genomics. AI algorithms can analyze vast genomic datasets to identify mutations and variations that might influence an individual’s response to certain treatments. For example, in oncology, AI helps in identifying specific genetic markers that are susceptible to targeted cancer therapies. This approach increases the efficacy of the treatment and minimizes the risk of adverse reactions, ensuring a more effective and safer treatment plan for the patient.
  A prime example of this application is a study focusing on nonmuscle invasive urothelial carcinoma, a type of bladder cancer known for its high recurrence risk [43]. In this study, researchers employed a machine learning algorithm to analyze genomic data from patients at their initial presentation. They aimed to identify genes most predictive of recurrence within five years following transurethral resection of the bladder tumor. The study involved whole-genome profiling of 112 frozen nonmuscle invasive urothelial carcinoma specimens using Human WG-6 BeadChips. A genetic programming algorithm was then applied to evolve classifier mathematical models for outcome prediction. The process involved cross-validation-based resampling and assessing gene use frequencies to pinpoint the most prognostic genes. These genes were subsequently combined into rules within a voting algorithm to predict the likelihood of cancer recurrence. Of the genes analyzed, 21 were identified as predictive of recurrence. Further validation through the quantitative polymerase chain reaction was conducted on a subset of 100 patients. The results were promising: a five-gene combined rule using the voting algorithm showed 77% sensitivity and 85% specificity in predicting recurrence in the training set. Additionally, a three-gene rule was developed, offering 80% sensitivity and 90% specificity in the training set for recurrence prediction.
2. Predictive analytics in drug development: AI also plays a crucial role in drug development, particularly in predicting how different patients will respond to a drug. By analyzing historical data from clinical trials and patient records, AI models can predict the effectiveness of drugs on various demographic groups [44,45]. This predictive power is invaluable in designing clinical trials and in developing drugs that are more effective for specific patient populations
  In recent years, AI has made remarkable strides in drug development. Exscientia introduced the first AI-designed drug molecule for clinical trials in early 2020 [46]. DeepMind’s AlphaFold then achieved a breakthrough in July 2021 by predicting structures for over 330,000 proteins, including the entire human genome. In 2022, Insilico Medicine started Phase I trials for an AI-discovered molecule, a process significantly faster and more cost-effective than traditional methods. By 2023, AbSci had innovated in creating antibodies using generative AI, and Insilico Medicine saw an AI-designed drug receive FDA Orphan Drug Designation, with Phase II trials planned shortly thereafter. These milestones mark a transformative era in AI-driven drug discovery.
  AI’s application extends to the identification of novel proteins or genes as potential disease targets, with systems capable of predicting the 3D structures of these targets using deep learning [47]. AI is also revolutionizing molecular simulations and the prediction of drug properties such as toxicity and bioactivity, enabling high-fidelity simulations that can be run entirely in silico [44]. Moreover, AI is shifting the paradigm of traditional drug discovery from screening large libraries of molecules to generating novel drug molecules from scratch [48]. This approach can enhance the efficiency of the drug discovery process and can lead to the development of novel therapies.

The growing industry interest in AI-enabled drug discovery is evident from the substantial investments flowing into the sector. The promise of lower costs, shorter development timelines, and the potential to treat currently incurable conditions positions AI as an important tool in the future of drug development.

The advances of AI in drug development underscore the necessity for legal and policy frameworks to adapt to these rapid technological changes, ensuring the continued assurance of drug safety and efficacy while harnessing the full potential of AI in healthcare.

3. Customizing treatment plans: AI systems are adept at integrating and analyzing various types of health data—from clinical records and lab results to lifestyle information and environmental factors. This capability allows healthcare providers to create more refined and comprehensive treatment plans [49]. For instance, in managing chronic diseases like diabetes, AI can analyze data from wearable devices, diet logs, and blood sugar readings to recommend personalized lifestyle and medication adjustments for better disease management [50].
4. AI in mental health: In the field of mental health, AI is used to personalize treatment approaches. By monitoring patterns in speech [51], behavior [52], and social media activity [53,54], AI tools can help in identifying the onset of mental health issues and suggest interventions tailored to the individual’s unique situation. This personalized approach is crucial in mental health, where treatment efficacy can vary significantly from person to person.

In future research and development within mental health treatment, a promising direction is the integration of AI systems with emotional intelligence [55]. Such systems could be crucial in early detection and intervention of mental health disorders by analyzing speech and behavior patterns for signs of conditions like depression or anxiety. Further exploration into personalizing therapy using AI could lead to more individualized and effective care.

Addressing accessibility is also crucial; AI-powered chatbots or virtual assistants can provide immediate support, overcoming barriers to traditional mental health services. Moreover, incorporating AI to assist therapists in real time during sessions could significantly enhance the effectiveness of therapy. Focusing on these aspects can transform mental health care into a more empathetic, accessible, and personalized practice, ultimately improving patient outcomes and support.

5. Key insights: While the integration of AI into personalized medicine offers transformative potential, it also presents a spectrum of challenges that must be addressed. Beyond data privacy and algorithmic bias, significant concerns include interoperability and data integration across diverse healthcare systems [56], ensuring AI systems are compliant with regulatory and ethical standards, and establishing their clinical validity and reliability [57].

Moreover, health equity remains a critical challenge, as AI must be accessible and beneficial to all population segments, avoiding disparities in healthcare [58]. The scalability and generalization of AI systems to various patient demographics and healthcare environments is also essential. Equally important is the training and acceptance of these tools among healthcare professionals. While AI may excel in certain diagnostic tasks, it serves as a valuable tool that enhances the capabilities of healthcare professionals rather than replacing human judgment entirely. Therefore, the integration of AI into healthcare workflows should be viewed as a symbiotic relationship, ultimately leading to improved patient outcomes. Additionally, cost considerations and effective resource allocation pose challenges in implementing AI solutions in healthcare settings [59].

## 3. AI in Hospital Operations and Management

In the complex and dynamic environment of hospitals and clinics, efficient operations and management are crucial for delivering quality healthcare. The integration of AI into these aspects creates a new era in healthcare management. This section explores how AI is being leveraged to revolutionize hospital operations, enhancing efficiency, reducing costs, and improving patient care. We will explore three primary areas: AI’s role in optimizing logistics and resource management, its application in automating administrative tasks, and its contribution to improving patient flow and scheduling.

Table 2 summarizes the transformative applications of AI in hospital operations and management.

### 3.1. AI for Hospital Logistics and Resource Management

Effective logistics and resource management are vital for the smooth functioning of any healthcare facility. AI technologies are playing an increasingly significant role in optimizing these aspects, leading to more efficient and cost-effective operations:

1. Inventory management: AI systems are being used to predictively manage inventory in hospitals [60,61]. By analyzing usage patterns, patient inflow, and other relevant data, AI can forecast the need for medical supplies, medications, and equipment. This predictive capability ensures that hospitals maintain optimal stock levels, reducing wastage and ensuring the availability of critical supplies when needed.
2. Facility management: AI also contributes to the efficient management of hospital facilities. For example, AI-powered systems can control heating, ventilation, and air conditioning (HVAC) systems more efficiently, reducing energy costs while maintaining a comfortable environment for patients and staff [62]. Additionally, AI can help in the predictive maintenance of hospital equipment, identifying potential issues before they lead to breakdowns, thus minimizing downtime and repair costs [63].
3. Resource allocation: One of the most substantial applications of AI in hospital management is in the optimization of resource allocation [64]. AI algorithms can analyze complex datasets, including patient admissions, staff availability, and operational capacities, to optimize the allocation of human and material resources. This includes scheduling surgeries and medical procedures in a manner that maximizes the utilization of operating rooms and medical staff, while minimizing patient wait times [65].
4. Supply chain optimization: AI enhances supply chain operations in hospitals by analyzing trends and automating ordering processes [66,67]. It can anticipate supply chain disruptions and suggest alternative solutions, ensuring that the hospital’s operations are not affected by external supply chain challenges. In emergency situations or during health crises, AI systems play a crucial role in managing logistics and resources [68]. They can quickly analyze the situation, predict the resources required, and assist in the efficient distribution of these resources where they are needed most.

In conclusion, AI’s role in hospital logistics and resource management is multifaceted and profoundly impactful. By automating and optimizing these critical aspects, AI can bring about operational efficiencies and enhance the overall quality of patient care. As AI technology continues to advance, its potential to further revolutionize hospital operations and management is vast, opening new avenues for innovation in healthcare delivery.

### 3.2. Automating Administrative Tasks with AI

This subsection examines how AI is being utilized to streamline administrative processes, thereby reducing the workload on healthcare staff and improving overall service delivery:

1. Patient data management: AI plays an important role in managing vast amounts of patient data [69]. AI systems can organize, categorize, and process patient records, appointments, and treatment histories with high efficiency and accuracy. These systems can also extract relevant information from unstructured data, such as doctor’s notes, making it easier for healthcare providers to access and analyze patient information. For example, a study utilized AI and natural language processing (NLP) to analyze electronic medical records (EMRs), focusing on uncoded consultation notes for disease prediction [70]. Techniques like bag of words and topic modeling were applied, along with a method to match notes with a medical ontology. This approach was particularly tested for colorectal cancer. The study found that the ontology-based method significantly enhanced predictive performance, with an AUC of 0.870, surpassing traditional benchmarks. This highlights AI’s potential in extracting useful information from EMR’s unstructured data, improving disease prediction accuracy.
2. Billing and claims processing: AI algorithms can also be used to automate billing and insurance claims processing. They can quickly analyze and process claims data, identify errors or inconsistencies, and ensure that billing is accurate and compliant with relevant regulations [71]. This not only speeds up the reimbursement process but also reduces the likelihood of billing errors, leading to improved financial operations and patient satisfaction. For example, a study in the insurance sector utilized machine learning to improve loss reserve estimation accuracy, crucial for financial statements [72]. Moving away from traditional macro-level models, this approach used individual claims data, integrating details about policies, policyholders, and claims. The method addressed the challenge of right-censored variables by creating tailored datasets for training and evaluating the algorithms. Compared to the conventional chain ladder method, this AI-driven approach showed notable improvements in accuracy, evidenced by a real case study with a Dutch loan insurance portfolio.
3. Scheduling appointments: AI-driven scheduling systems are revolutionizing the way appointments are managed in healthcare settings [73]. These systems can analyze patterns in appointment bookings and cancellations to optimize the scheduling of patients. By predicting peak times and adjusting appointments accordingly, AI helps in reducing wait times and improving patient flow. For example, a project aimed at reducing outpatient MRI no-shows effectively utilized AI predictive analytics [74]. In this quality improvement initiative, over 32,000 anonymized outpatient MRI appointment records were analyzed using machine learning techniques, specifically an XGBoost model, a decision tree-based ensemble algorithm. This approach achieved notable results; the model’s predictive accuracy was demonstrated by an ROC AUC of 0.746 and an optimized F1 score of 0.708. When implemented alongside a practical intervention of telephone call reminders for patients identified as high-risk for no-shows, the no-show rate decreased from 19.3% to 15.9% over six months. In another study, a data-driven approach was used to optimize appointment scheduling and sequencing, especially in environments with uncertain service durations and customer punctuality [75]. Leveraging a novel method based on infinite-server queues, the study developed scalable solutions suitable for complex systems with numerous jobs and servers. Tested using a comprehensive dataset from a cancer center’s infusion unit, this approach significantly improved operational efficiency. The results showed a consistent reduction in costs—combining waiting times and overtime—by 15% to 40%, demonstrating the effectiveness of AI-based strategies in optimizing appointment scheduling.
4. Document management and processing: AI technologies are adept at automating the processing of various documents, including consent forms, admission forms, and medical reports [76]. By using natural language processing (NLP) and machine learning, AI can quickly parse through documents, extract relevant information, and categorize them appropriately. This automation reduces the administrative burden on staff and speeds up document processing.
5. Automated communication and reminders: A notable application of AI in healthcare is the optimization of information extraction from electronic health records (EHRs), particularly from scanned documents. A study demonstrated this by successfully extracting sleep apnea indicators from scanned sleep study reports using a combination of image preprocessing techniques and natural language processing (NLP) [77]. By employing methods like gray-scaling and OCR with Tesseract, followed by analysis through advanced models like ClinicalBERT, the study achieved high accuracy rates (over 90%) in identifying key health metrics.
6. Automated communication and reminders: AI-powered chatbots and virtual assistants are increasingly used for patient communication. They can handle routine inquiries, provide information about services, and send reminders for upcoming appointments or medication schedules. This not only enhances patient engagement but also frees up staff to focus on more critical tasks.
  An example of this application is seen in the ChronologyMD project [78], which utilized AI to improve eHealth communication programs. The project addressed major deficiencies in existing eHealth communication strategies, which often failed to fully engage audiences and sometimes even negatively impacted the delivery of crucial health information. By strategically employing AI, the ChronologyMD project succeeded in making health communication more engaging, relevant, and actionable. Additionally, it led to increased exposure to relevant messages, reduced the workload of healthcare staff, and improved the overall efficiency of the program while minimizing costs.
7. Data security and compliance: AI systems contribute significantly to data security and compliance in healthcare [79]. They can monitor and analyze data access patterns to detect and prevent unauthorized access or breaches. Additionally, AI can ensure that administrative processes are compliant with healthcare regulations, such as HIPAA, thereby safeguarding patient privacy.
  Building on this, recent research has explored the role of AI in ensuring compliance with the General Data Protection Regulation (GDPR), crucial for data controllers [80]. This study aimed to bridge gaps in compliance checking through a two-pronged approach: firstly, by conceptualizing a framework for document-centric compliance checking in the data supply chain, and secondly, by developing methods to automate the compliance checking of privacy policies. The study tested a two-module system, where the first module uses natural language processing (NLP) to extract data practices from privacy policies, and the second module encodes GDPR rules to ensure the inclusion of all mandatory information. The results demonstrated that this text-to-text approach was more effective than local classifiers, capable of extracting both broad and specific information with a single model. The system’s effectiveness was validated on a dataset of 30 privacy policies, annotated by legal experts.

In summary, automating administrative tasks with AI significantly enhances the efficiency and accuracy of hospital operations. It allows healthcare professionals to focus more on patient care rather than administrative duties, leading to improved healthcare delivery. As AI technology continues to evolve, it could progress from automating tasks to personalizing patient interactions through emotional intelligence and cultural awareness, ultimately aiming to provide a more holistic and supportive care experience.

### 3.3. AI in Patient Flow and Scheduling Optimization

The effective management of patient flow and scheduling is a critical component of hospital operations, impacting both patient satisfaction and healthcare delivery efficiency. The integration of AI in this domain has shown significant promise in optimizing these processes:

1. Optimizing patient flow: AI algorithms are particularly adept at analyzing patterns in patient admissions, discharges, and transfers, enabling more efficient patient flow throughout the hospital [65,81]. By predicting high-demand periods, AI can assist in preemptively allocating resources such as beds, staff, and equipment to meet patient needs. For instance, AI systems can forecast daily or seasonal fluctuations in patient admissions, allowing hospitals to adjust staffing levels and bed availability accordingly [82]. This proactive approach reduces bottlenecks, minimizes wait times, and enhances the overall patient experience.
2. Dynamic scheduling systems: AI-driven scheduling systems revolutionize the way appointments and procedures are organized. These systems can analyze multiple variables, including healthcare provider availability, patient preferences, and urgency of care, to create optimal schedules. By doing so, they reduce appointment no-shows and last-minute cancellations, maximizing the utilization of healthcare professionals’ time. Moreover, these AI systems can adapt in real time to changes, such as emergency cases, by rescheduling non-urgent appointments without significant disruptions [83].
  In a study aimed at improving outpatient department efficiency and patient satisfaction, researchers developed an innovative appointment scheduling system based on a Markov decision process model, incorporating patient preferences to maximize satisfaction [84]. Adaptive dynamic programming algorithms were utilized to overcome the complexity of scheduling, dynamically adjusting to patient preferences and continuously improving appointment decisions. The system’s performance was evaluated through various experiments, which demonstrated optimal convergence behavior and accuracy.
3. Reducing waiting times: One of the critical benefits of AI in patient flow is the reduction in waiting times in emergency departments and outpatient clinics. AI can predict patient inflow and identify potential delays, allowing hospital staff to take proactive measures to manage patient wait times effectively [85,86]. For emergency departments, this means better triage processes and quicker allocation of patients to the appropriate care.
  Utilizing machine learning algorithms, a recent study predicted patient waiting times before consultation and throughput time in an outpatient clinic, aiming to enhance patient satisfaction by providing more accurate wait time information [87]. The study employed random forest and XGBoost algorithms, analyzing input variables such as gender, day and time of visit, and consultation session. The study achieved high accuracy (86–93%) in predicting wait and throughput times in an outpatient clinic using machine learning models with novel input variables.
4. Enhancing patient experience: AI systems can also improve the overall patient experience by providing accurate information about appointment times, wait periods, and treatment schedules [88]. This transparency helps in managing patient expectations and reduces anxiety associated with medical appointments and procedures.
  In a recent study, a machine learning model was developed to predict patient responses to the “Doctor Communications” domain of the Hospital Consumer Assessment of Healthcare Providers and Systems survey, using data from a tertiary care hospital (2016–2020) [89]. The random forest algorithm effectively predicted patient responses about doctors’ courtesy, explanation clarity, and attentiveness. The model achieved an AUC of 88% for these doctor communication survey questions.
5. Integrating with telehealth: In the era of digital health, AI in scheduling extends beyond in-person appointments to include telehealth services. AI systems can effectively schedule and manage virtual consultations, ensuring that patients receive timely care without the need to physically visit the healthcare facility, which is particularly beneficial for routine follow-ups or during health crises like pandemics [90].

In conclusion, AI’s role in optimizing patient flow and scheduling in hospitals and clinics is profoundly transformative, offering significant enhancements in operational efficiency, reduced waiting times, and improved patient experiences. As an important element in modernizing healthcare delivery, AI-driven optimization strategies are increasingly crucial. Looking to the future, AI technology is poised for further evolution, with potential advances including real-time adaptive scheduling algorithms, deeper integration with electronic health records for more personalized patient care, and the use of predictive analytics for anticipating patient demand and resource allocation.

## 4. AI in Medical Imaging and Diagnostics

The integration of AI into medical imaging and diagnostics marks a transformative development in healthcare. This section examines how AI is reshaping the fields of radiology and pathology, bringing unprecedented levels of accuracy and efficiency. We will explore AI’s expanding role in enhancing diagnostic processes and review specific examples of AI systems in imaging technologies such as MRI and CT scans.

### 4.1. AI’s Role in Radiology and Pathology

AI’s impact on radiology and pathology has been profound, revolutionizing the way medical images are analyzed and interpreted.

In radiology, AI algorithms, particularly those based on deep learning, are increasingly being used to analyze radiographic images. These AI models are trained on vast datasets of X-rays [91], MRIs [92], CT scans [93], and other imaging modalities [94], enabling them to detect abnormalities such as tumors, fractures, and signs of diseases like pneumonia or brain bleeds with high precision. In many cases, AI can highlight subtle findings that may be overlooked by the human eye, thus serving as an invaluable tool for radiologists. For example, a recent study introduced an anatomy-aware graph convolutional network (AGN) tailored for mammogram mass detection, enabling multi-view reasoning akin to radiologists’ natural ability [95]. This AGN, significantly outperforming current methods on benchmarks, involves modeling relations in ipsilateral and bilateral mammogram views, and its visualization results offer interpretable cues crucial for clinical diagnosis.

AI in radiology is not only about detecting abnormalities; it also helps in quantifying disease progression [96], assessing response to treatment [97], and predicting patient outcomes [98]. For example, in cancer treatment, AI can measure the size and growth of tumors over time, providing crucial information for treatment planning [99].

The field of pathology has also seen significant advancements with the integration of AI [100]. Digital pathology, where slides are scanned and analyzed by AI algorithms, has enabled more accurate and faster diagnosis of diseases. AI excels in pattern recognition, which is essential in identifying markers of diseases in tissue samples. This is particularly impactful in the diagnosis of cancers, where AI can assist pathologists in spotting cancerous cells, often with greater accuracy and speed than traditional methods. As an example, deep learning neural networks have significantly advanced molecular diagnostics in clinical oncology, leading to a new era in digital pathology and precision medicine [101]. This advancement holds significant promise particularly for resource-limited settings. For example, in India, an AI-powered software has been used to analyze key molecular markers in endoscopic images, enabling more precise diagnoses of gastric cancer, potentially paving the way for personalized treatment approaches [102].

AI’s contribution to pathology extends beyond disease detection. It also includes predicting disease aggressiveness and patient prognosis, helping pathologists make more informed decisions about patient care. For example, an AI model utilizing MRI scans accurately predicts the aggressiveness of soft tissue sarcomas with an average accuracy of 84.3% and sensitivity of 73.3%, providing valuable insights as a second expert opinion for clinicians prior to biopsy, presenting a novel approach for rare pathology diagnosis [103].

In summary, AI’s role in radiology and pathology is transformative, offering advanced diagnostic capabilities. However, this progress invites critical considerations, such as the need for ongoing training for medical professionals to effectively integrate AI tools, and continuous evaluation of AI systems to ensure they complement rather than replace human expertise. Future advancements should aim to harmonize AI technology with clinical practice, ensuring it remains a supportive tool that enhances, rather than overshadows, the critical role of medical professionals.

### 4.2. Enhancing Accuracy and Efficiency in Diagnostic Processes

The incorporation of AI into diagnostic processes is a game-changer in healthcare, notably enhancing both accuracy and efficiency. This subsection considers the various ways in which AI is achieving these improvements and the impact it has on the overall diagnostic workflow:

1. Improving diagnostic accuracy: AI algorithms, particularly those based on deep learning, have demonstrated remarkable accuracy in diagnosing diseases from medical images and test results. These systems are trained on vast datasets, allowing them to recognize patterns and anomalies that might be imperceptible to the human eye. For example, in dermatology, AI systems trained on images of skin lesions have shown the ability to detect skin cancers, such as melanoma, with a level of precision comparable to that of experienced dermatologists [104].
2. Reducing diagnostic errors: One of the key benefits of AI in diagnostics is its potential to reduce errors [105]. Misdiagnosis and missed diagnoses are significant concerns in medicine, often leading to delayed or inappropriate treatment. AI systems provide a level of consistency and attention to detail that is challenging for humans to maintain over long periods, thus reducing the likelihood of such errors.
3. Speeding up diagnostic processes: AI significantly speeds up the diagnostic process. Analyzing medical images or test results, tasks that would take a healthcare professional considerable time, can be performed by AI in a fraction of the time. This rapid analysis is particularly beneficial in urgent care situations, where quick decision-making is critical. For instance, AI algorithms can quickly analyze CT scans of stroke patients to identify blockages or bleeding in the brain, enabling faster initiation of life-saving treatments [106].
4. Automated reporting and documentation: AI not only automates reporting and documentation in diagnostic processes [107] but also enhances the quality of these processes. While AI systems generate preliminary reports from image analysis for radiologist review, streamlining workflow and reducing administrative burden, a recent study has furthered this efficiency by consolidating existing ML reporting guidelines [108]. This study, after an extensive review of 192 articles and expert feedback, created a comprehensive checklist encompassing 37 reporting items for prognostic and diagnostic ML studies. This effort in standardizing ML reporting is pivotal in improving the quality and reproducibility of ML modeling studies, complementing AI’s role in simplifying diagnostic reporting.
5. Integrating diagnostic data: AI excels in integrating and analyzing data from various sources. In the case of complex diseases, AI can combine information from imaging, laboratory tests, and patient histories to provide a more comprehensive diagnostic insight [109]. This integration is particularly valuable in diagnosing complex conditions like autoimmune diseases or in cases where symptoms are ambiguous.
  As an example, a scoping review focused on AI techniques for fusing multimodal medical data, particularly EHR with medical imaging, to develop AI methods for various clinical applications [110]. The review analyzed 34 studies, observing a workflow of combining raw data using ML or DL algorithms for clinical outcome predictions. It found that multimodality fusion models generally outperform single-modality models, with early fusion being the most commonly used technique. Neurological disorders were the dominant category studied, and conventional ML models were more frequently used than DL models. This review provides insights into the current state of multimodal medical data fusion in healthcare research.

In conclusion, AI’s significant role in improving diagnostic accuracy and efficiency is transforming healthcare, delivering faster and more precise diagnoses. However, a critical concern is that these AI systems are often primarily designed for specific groups, which can lead to disparities in healthcare. Future advancements should emphasize the development of more inclusive AI models that cater to a broader patient demographic, ensuring equitable healthcare improvements across all populations.

### 4.3. The Role of Hardware Acceleration in AI-Powered Diagnostics

The previous sections explored how AI is revolutionizing medical imaging and diagnostics by enhancing accuracy and efficiency. However, this transformation hinges on the immense processing power required to analyze large medical datasets of X-rays, MRIs, and CT scans, along with the complex AI algorithms used for tasks like image recognition and disease detection. This is where hardware acceleration steps in, acting as a powerful engine that fuels AI-powered diagnostics [111].

Hardware accelerators are specialized components within a computer system designed to offload and expedite specific computing tasks typically handled by the main processor (CPU). While CPUs are versatile, they may not always be the most efficient for computationally intensive AI workloads. Hardware accelerators, on the other hand, are optimized for these tasks, offering significant performance boosts.

Several types of hardware accelerators are well suited for AI-powered diagnostics [112]:

1. Graphics Processing Units (GPUs): Originally designed for computer graphics rendering, GPUs excel at parallel processing, making them ideal for handling the massive datasets and complex calculations involved in AI algorithms. In the medical image analysis domain, GPUs can be used to accelerate basic image processing operations such as filtering and interpolation. Additionally, GPUs can enhance the operation of different AI algorithms used in medical imaging tasks like image registration, image segmentation, image denoising, and image classification [113].
2. Tensor Processing Units (TPUs): Custom-designed chips like TPUs, pioneered by companies like Google, are specifically optimized for high-performance deep learning inference, a key technique used in medical image analysis. TPUs offer significant speed advantages over CPUs for tasks like image recognition and classification. For example, researchers implemented a system for glaucoma diagnosis using both edge TPUs and embedded GPUs [114]. While both achieved fast image segmentation and classification for real-time diagnosis support, the study found that TPUs consumed significantly less energy compared to GPUs. This makes TPUs a more attractive option for battery-powered medical devices used in edge computing scenarios.
3. Field-Programmable Gate Arrays (FPGAs): These versatile chips offer flexibility for hardware customization. Unlike pre-designed GPUs and TPUs, FPGAs can be programmed to perform specific AI algorithms, potentially leading to highly optimized solutions for certain diagnostic tasks. However, programming FPGAs requires specialized expertise. For instance, researchers have proposed a MobileNet accelerator designed specifically for FPGAs that focuses on minimizing on-chip memory usage and data transfer, making it ideal for low-power devices [115]. They achieve this by using two configurable modules for different convolution operations and a new cache usage method. Their implementation demonstrates real-time processing with low memory usage, making FPGAs a viable option for running efficient CNNs in auxiliary medical tasks on portable devices.
4. Application-Specific Integrated Circuits (ASICs): When dealing with a well-defined AI algorithm in a specific diagnostic application, ASICs can be designed to offer the ultimate performance [116]. Engineered for a single task, ASICs provide unparalleled efficiency and processing speed for that specific function. However, the lack of flexibility limits their application to well-established and unchanging algorithms.

By leveraging hardware acceleration, AI-powered diagnostics can achieve several benefits: faster processing for near-real-time analysis of medical images, leading to quicker and potentially life-saving interventions; improved accuracy through the ability to perform intricate image analysis, potentially leading to a higher degree of disease detection; and enhanced efficiency by streamlining the diagnostic process, allowing radiologists and clinicians to analyze more images in a shorter timeframe.

It is important to note that these benefits extend beyond medical imaging, with hardware acceleration playing a crucial role in other AI health tasks such as analyzing genetic data for personalized medicine or processing real-time sensor data from wearable devices for remote patient monitoring [117].

### 4.4. Examples of AI Systems Used in Imaging

AI has made significant contributions in the field of medical imaging, with various AI systems being developed and used for analyzing images from MRI, CT scans, and other modalities. This subsection highlights some notable examples of these AI systems, showcasing their capabilities and the impact they have on diagnostic imaging. An overview of AI applications in medical imaging is also presented in Table 3.

1. AI in MRI analysis: AI applications in MRI analysis are versatile, encompassing the detection of brain abnormalities, tumors, strokes, neurodegenerative diseases, musculoskeletal injuries, cardiac conditions, and liver and abdominal organ pathologies, as well as evaluating breast and prostate cancers, demonstrating its broad utility in diagnosing a wide range of medical conditions [122,123]. In addition, deep learning is now playing a key role in accelerating the MRI acquisition process [92].
  An example of AI application in MRI is an AI system developed for detecting brain abnormalities [118]. This system uses a deep CNN to analyze MRI images and can identify conditions such as tumors, strokes, and neurodegenerative diseases. The AI not only detects these abnormalities but also helps in quantifying the volume of affected areas, which is vital for treatment planning and monitoring disease progression. Another example is the application of AI in the interpretation of breast cancer. CNNs are employed to extract features from MRI breast scans, and alongside classifiers, they effectively detect the presence of cancer, showcasing the potential of AI in enhancing diagnostic accuracy in breast cancer detection [124].
  AI systems are increasingly used for the automated segmentation of images in radiology [125]. These systems can differentiate and label various anatomical structures in the images, such as organs and tissues, aiding radiologists in diagnosis and in planning surgeries or treatments. For example, a study introduced a 4D deep learning model, combining 3D convolution and LSTM, for the precise segmentation of hepatocellular carcinoma (HCC) lesions in dynamic contrast-enhanced MRI images [126]. Utilizing both spatial and temporal domain information from multi-phase images, the model significantly improved liver tumor segmentation performance, achieving superior metrics compared to existing models and offering a comparable performance to the state-of-the-art nnU-Net model with reduced prediction time.
  AI is also being adapted for pediatric imaging, addressing the unique challenges presented by the varying sizes and developmental stages of pediatric patients [127]. AI systems in this domain are tailored to recognize and interpret patterns specific to children, aiding in the diagnosis of congenital and developmental conditions. For instance, in pediatric imaging for focal epilepsy, a deep CNN model was introduced, excelling in tract classification and identifying critical white matter pathways with 98% accuracy [128]. This model effectively predicted surgical outcomes and postoperative language changes, showcasing its potential to enhance preoperative evaluations and improve surgical precision in children.
2. AI for CT scan interpretation: AI applications in CT scan interpretation span detecting lung nodules, identifying fractures and hemorrhages, assessing stroke severity, and characterizing tumor progression. One innovative AI application in CT imaging is in the rapid identification of pulmonary embolisms [119]. The AI system processes CT pulmonary angiograms to detect blood clots in the lungs with high accuracy, often faster than traditional methods. This speed is critical in emergency situations, where timely intervention can be life-saving. As another example, Google’s AI, in collaboration with researchers from Northwestern University, NYU-Langone Medical Center, and Stanford Medicine, has developed a CT scan model that diagnoses lung cancer with accuracy equal to or surpassing six radiologists [129]. This model analyzes 3D volumetric scans to predict malignancy and detect subtle lung nodules, viewing the lungs as a single 3D object and comparing scans over time to track lesion growth. Tested on over 45,800 de-identified chest CT screenings, it detected 5% more cancer cases and reduced false positives by over 11% compared to traditional radiologist evaluations, demonstrating significant potential for enhancing lung cancer diagnosis.
3. AI in X-ray analysis: AI is revolutionizing X-ray analysis across various medical fields. Take mammography, for instance, AI is transforming breast cancer screening by enhancing image analysis for tumor detection, improving accuracy in identifying benign and malignant lesions, and reducing false positives and negatives, thereby streamlining the diagnostic process for early and effective treatment [130]. These systems analyze mammograms to identify signs of cancerous lesions, with some AI models demonstrating the ability to detect cancers that were initially missed by radiologists. By serving as a second reviewer, these AI systems enhance the accuracy of breast cancer screening. A recent study demonstrated that cmAssist™, an AI-based CAD algorithm based on multiple custom deep learning-based networks, significantly enhanced radiologists’ sensitivity in breast cancer detection [120]. Analyzing 122 mammograms with a blend of false negatives and BIRADS 1 and 2 ratings, radiologists showed a notable improvement in cancer detection rates (CDRs) by an average of 27% when using cmAssist, with a minimal increase in false positives. This marked improvement underscores the potential of AI-CAD software in improving accuracy and sensitivity in breast cancer screening.
4. AI in ultrasound: AI is significantly impacting various applications of ultrasound. In cardiac imaging, for example, AI systems are used to analyze images from echocardiography scans to assess cardiovascular function [131]. They can measure parameters such as the ejection fraction, which indicates how well the heart is pumping blood, and detect structural abnormalities of the heart. This information is crucial in diagnosing and managing heart diseases. For example, a study evaluating a novel AI for automated left ventricular ejection time calculation in echocardiography showed high accuracy, closely correlating with cardiac MRI results [121]. The AI, which demonstrated lower bias and greater reliability especially in challenging cases, outperformed conventional methods. This algorithm is based on a patented CNN, though specific details of its architecture and training process remain proprietary. This underscores the algorithm’s potential in reducing user-dependent variability and enhancing the clinical utility of echocardiography.

In conclusion, these examples illustrate the diverse and impactful applications of AI in medical imaging. By enhancing the accuracy, speed, and efficiency of image analysis, AI systems are proving to be invaluable assets in diagnostic radiology, ultimately leading to better patient care and outcomes. As AI technology continues to advance, its applications in medical imaging are expected to broaden, further transforming the field of radiology.

## 5. AI in Patient Care and Monitoring

The rise of AI in healthcare marks a paradigm shift, promising a future of more efficient and effective patient care and monitoring. This section explores how AI is enhancing patient care through innovative technologies and personalized approaches. The focus is on three key areas: AI-powered wearable devices for continuous monitoring, the impact of virtual nursing assistants, and AI’s role in telemedicine and remote patient engagement. These applications of AI are transforming the way patient care is administered and are empowering patients with more control over their health and wellness. Table 4 presents a summary of AI powered technologies for patient care and monitoring covered in this section. These topics are further discussed in the following:

### 5.1. AI-Powered Wearable Devices for Continuous Monitoring

AI-powered wearables mark a breakthrough in patient monitoring, blending convenience with real-time analysis of vital signs like heart rate, blood pressure, blood glucose, and oxygen saturation. They can also capture additional physiological data like electroencephalography (EEG), electrical activity of the heart (electrocardiography, ECG), and peripheral physiological signals like photoplethysmography (PPG), providing a more comprehensive picture of a patient’s health. Especially valuable for managing chronic conditions, these devices provide timely alerts for crucial interventions, such as notifying diabetic patients of blood sugar levels to prevent critical episodes [132].

One of the most impactful aspects of these wearables is their ability to analyze collected data and predict potential health issues before they become serious. Utilizing AI algorithms, these devices can detect patterns or anomalies in health data indicative of emerging problems. For instance, wearables can analyze heart rate variability [133], other cardiac markers [134], and sleep patterns [135] to predict the risk of heart conditions and sleep disorders, facilitating early preventive measures. For example, a novel deep learning framework based on a hybrid CNN-LSTM model forecasts sleep apnea occurrence from single-lead ECG with an accuracy of up to 94.95% when validated on 70 sleep recordings [135]. This approach utilizes ECG R-peak amplitudes and R-R intervals, making it suitable for wearable sleep monitors to manage sleep apnea effectively.

AI-powered wearables significantly enhance patient engagement by offering insights into health metrics and progress, encouraging active health management [136]. These devices, often paired with companion apps, provide personalized recommendations for lifestyle changes, medication adherence, and exercise based on the patient’s health data [137]. Additionally, they are increasingly being used for sleep monitoring, offering valuable data on sleep patterns and quality [138]. This feature aids in identifying sleep-related issues, allowing for targeted interventions that can improve overall well-being and health management.

While AI-powered wearables hold promise for revolutionizing patient care, they face specific challenges from data collection to model deployment [139]. Collecting sufficient, reliable data for training, especially in healthcare, is difficult due to high costs and the complexity of ensuring data reliability. Selecting the most effective features and frameworks and evaluating and deploying the best ML models add layers of complexity, compounded by the necessity for models to generalize well across diverse personal features. Wearable device developers must also navigate the selection of deployment options, balancing the advantages of on-device computing against the limitations of power consumption, storage, and computational power. Addressing these challenges involves a careful trade-off between model accuracy and the practical constraints of wearable technology, requiring innovations in model design, data processing, and system integration to optimize the clinical impact and user acceptance of wearable ML applications.

### 5.2. Virtual Nursing Assistants

Virtual nursing assistants, powered by AI, are transforming healthcare by offering continuous patient support and enhancing the efficiency of healthcare services [140]. These systems provide round-the-clock assistance, including health-related queries, medication reminders, and appointment scheduling, thereby supporting both patients and healthcare professionals. For example, AI-driven voice technology, through chatbots on mobile phones and smart speakers, enhances patient management and healthcare workflow, offering solutions for acute care triaging, chronic disease management, and telehealth services, particularly noted during the COVID-19 pandemic [141].

AI systems enhance patient engagement and education through personalized interactions, improving compliance with treatment plans and encouraging healthier lifestyle choices. A recent study in the Greater Toronto area on patient engagement in AI healthcare development educated diverse participants on AI before gathering their perspectives. The results indicated a strong desire for early and diverse patient involvement in AI development stages, emphasizing the critical role of patient education for meaningful engagement [142].

Additionally, they monitor health status and symptoms for those with chronic conditions, alerting healthcare providers when necessary to prevent complications and reduce hospital readmissions [143]. Virtual nursing assistants also collect and analyze patient data, offering insights into patient behavior and healthcare trends [144].

Despite their benefits, challenges such as data privacy, information accuracy, and ensuring they complement human care remain. With ongoing advancements in AI, virtual nursing assistants are expected to become more enhanced, promising a future of accessible, personalized, and efficient healthcare.

### 5.3. AI in Telemedicine and Remote Patient Engagement

The integration of AI into telemedicine and remote patient engagement is revolutionizing healthcare accessibility and effectiveness [145]. AI is enhancing telehealth platforms with advanced diagnostic and consultation services, enabling healthcare providers to diagnose patients remotely and personalize virtual consultations based on patient data [146]. AI-powered chatbots and virtual assistants facilitate patient interaction, offering support and streamlining the appointment process [147], while AI’s role in remote patient monitoring and predictive analytics supports proactive care for chronic conditions and anticipates potential health issues. For example, a study developed and evaluated PROSCA, an AI-based medical chatbot for prostate cancer education, involving ten men with suspicion of prostate cancer [148]. The chatbot effectively increased prostate cancer knowledge among 89% of its users, with all participants expressing a willingness to reuse and support chatbots in clinical settings, highlighting its potential in enhancing patient education and doctor–patient communication.

While AI integration into telemedicine offers enhanced capabilities for remote healthcare delivery, challenges including data privacy, system accuracy, and seamless healthcare system integration persist [149]. Despite these obstacles, AI’s incorporation into telemedicine remains crucial and offers a more accessible, personalized, and proactive healthcare future, where technology effectively narrows the distance between patients and providers, supported by physician-guided implementation and adherence to clinical practices.

## 6. Methodologies for Assessing AI Healthcare Solutions

Evaluating AI-based healthcare solutions requires a comprehensive approach that considers various aspects of performance, effectiveness, safety, and ethical considerations. In this section, we explore the methodologies employed to assess the viability and impact of AI technologies within healthcare settings.

### 6.1. Validation

Validation encompasses multiple stages, each crucial for ensuring the reliability and effectiveness of AI algorithms in healthcare, as elaborated below:

1. Algorithm validation: The successful integration of AI algorithms into healthcare hinges on their accuracy, reliability, and performance. This necessitates comprehensive testing using diverse datasets [150]. A critical challenge in this process is overfitting, where the algorithm performs well on the training data but fails to generalize to unseen data. To address this, techniques like cross-validation are employed [151]. Cross-validation involves splitting the training data into multiple folds and iteratively training the algorithm on a subset of folds while using the remaining folds for validation. This process helps assess how well the algorithm generalizes to new data and prevents overfitting. Beyond generalizability, AI in healthcare should be adaptable for personalized use. This means the algorithms should continuously learn from individual patient data to enable tailored treatment approaches. Rigorous assessment helps identify strengths, weaknesses, and areas for improvement, ultimately enhancing the reliability of AI-based healthcare solutions. Furthermore, validation on different patient groups is essential to address potential biases in the training data. Biases can lead to unfair and ineffective outcomes for certain demographics. By ensuring the algorithms perform consistently across diverse populations, we can ensure fairness and effectiveness for all.
2. Clinical validation: Clinical validation plays a crucial role in assessing the efficacy and safety of AI interventions [152]. Rigorous clinical trials and studies should be conducted to compare AI-based interventions with standard treatments or existing practices. These evaluations can encompass a range of study designs, including randomized controlled trials (RCTs), observational studies, or real-world evidence analyses. Through these studies, researchers can determine the effectiveness of AI technologies in improving patient outcomes and clinical decision-making. Furthermore, defining appropriate outcome measures is essential for assessing the impact of AI interventions on patient outcomes. Outcome measures such as mortality rates, disease progression, quality of life, and healthcare costs can be used to evaluate the effectiveness of AI technologies in improving healthcare delivery.

### 6.2. Interpretability and Usability

To earn trust and acceptance within the healthcare system, AI technologies must be interpretable, usable, and ethically sound. Interpretability ensures that AI models provide clear explanations for their decisions, fostering trust with clinicians who can understand the reasoning behind recommendations [153]. Usability focuses on the seamless integration of AI tools into existing workflows for all stakeholders. User-centered design principles, with active involvement from clinicians and patients throughout development, are crucial not only for usability but also for user engagement. This collaborative approach fosters a sense of ownership and trust in the AI solution, ultimately driving successful adoption and improved patient outcomes.

Furthermore, interpretability extends beyond simply understanding the “why” behind an AI decision. Explainability techniques like feature importance analysis, LIME (Local Interpretable Model-agnostic Explanations) [154], and SHAP (SHapley Additive exPlanations) [155] values can provide deeper insights into the model’s reasoning.

While interpretability and usability are crucial for the initial acceptance of AI solutions, user engagement plays a vital role in driving long-term trust and successful adoption [156]. User engagement refers to the ongoing interaction and positive user experience with the AI tool. User-centered design principles can promote engagement as follows:

1. Active stakeholder involvement: Throughout the development process, actively involving clinicians, patients, and other stakeholders provides valuable insights into their needs and expectations. This collaborative approach fosters a sense of ownership in the solution, leading to higher engagement.
2. Iterative development and feedback loops: Developing AI solutions is an iterative process. By incorporating user feedback throughout development cycles, researchers can refine the AI tool to better address user needs. This ongoing feedback loop not only improves usability but also strengthens user confidence and engagement.
3. User-friendly interfaces and clear visualizations: Designing clear and user-friendly interfaces is essential for user engagement. This includes presenting AI outputs in a way that is easy to understand and interpret, even for users with limited technical expertise. Additionally, providing clear visualizations of the AI’s reasoning can further enhance user trust and engagement.

### 6.3. Scalability and Continuous Improvement

Scalability refers to the ability of AI models to adapt and perform effectively across diverse healthcare settings, patient populations, and clinical scenarios [157]. An AI model trained in a large academic hospital, to be truly impactful, needs to adapt and deliver accurate results in smaller clinics with different patient populations and clinical scenarios. Scalability ensures AI solutions can be implemented and benefit a wider range of healthcare providers and patients.

Continuous improvement involves implementing mechanisms for ongoing monitoring, feedback collection, and iterative enhancement of AI solutions over time. This may include the following:

1. Post-market surveillance: Closely monitoring the performance of AI solutions after deployment in real-world settings to identify any unforeseen issues or areas for improvement [158].
2. Performance monitoring: Continuously tracking the effectiveness of the AI tool in achieving its intended outcomes [159]. These data can be used to identify areas where the AI can be further optimized.
3. Updating algorithms based on new data and insights: AI algorithms are not static. As new data become available, or as researchers gain a deeper understanding of the underlying problem, the algorithms can be updated to improve their performance and accuracy.

By prioritizing scalability and continuous improvement, researchers and developers should ensure the long-term success and sustainability of AI-based healthcare solutions in addressing evolving healthcare challenges.

## 7. Ethical Considerations and Challenges

**Figure 2:**
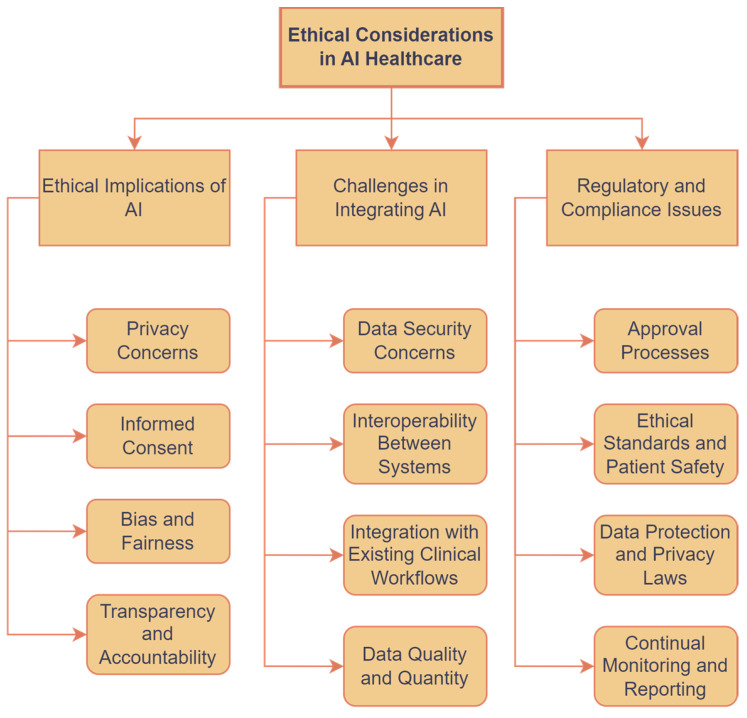
Navigating ethical considerations and challenges in healthcare AI.

As AI continues to enhance the healthcare sector, it brings significant ethical considerations and challenges. This section explores the complex ethical landscape surrounding the use of AI in healthcare. We will explore the implications of AI on privacy, consent, and bias, scrutinize the practical challenges in its integration, such as data security and interoperability, and discuss the evolving regulatory and compliance landscape. The integration of AI into healthcare raises fundamental questions about patient rights, data stewardship, and the equitable delivery of care, demanding a thoughtful and refined approach to its deployment. Figure 2 navigates the ethical considerations and challenges in healthcare AI. These topics are further discussed in the following sections:

### 7.1. Ethical Implications of AI in Healthcare

The ethical implications of AI in healthcare include various possibilities, including the following:

1. Privacy concerns: One of the foremost ethical concerns in AI healthcare is the privacy of patient data. AI systems require access to large datasets of patient information, which raises questions about the security and confidentiality of sensitive health data [160]. Ensuring that patient data used for AI applications are anonymized and securely stored is paramount. There is also a need for transparent policies regarding who has access to these data and for what purposes.
2. Informed consent: The issue of informed consent in AI healthcare is complex, necessitating clear communication with patients about the use of their data, especially with AI algorithms that may be challenging for non-experts to grasp. This includes detailing data sharing implications, potential benefits and risks associated with AI-driven healthcare, and the level of human oversight in AI decisions. More details on the use of informed consent forms for AI in medicine with a comprehensive guideline for emergency physicians can be found in [161].
3. Bias and fairness: AI systems are only as unbiased as the data they are trained on. There is a risk that AI algorithms may perpetuate existing biases present in healthcare data, leading to unfair treatment outcomes for certain groups [162]. For example, if an AI system is trained predominantly on data from a specific demographic, its accuracy might be lower for patients outside of that demographic. Ensuring that AI systems are developed and trained on diverse datasets is crucial to mitigate these biases. Moreover, the continuous monitoring and auditing of AI systems for biased outcomes are necessary to uphold fairness in healthcare delivery.
4. Transparency and accountability: Transparency in AI decision-making processes is a key ethical concern [163]. It is important for healthcare providers and patients to understand how AI systems make their recommendations. This transparency is essential for building trust in AI systems and for accountability [164]. In cases where AI-driven decisions impact patient care, it is crucial to have mechanisms in place to review and understand these decisions, particularly in the event of adverse outcomes. A recent study highlights the need for transparent and accountable AI systems in natural NLP to address the “black box” issue of deep learning models [165]. It introduces the Explaining and Visualizing CNNs for Text Information (EVCT) framework, which offers human-interpretable solutions for text classification with minimal information loss, aligning with recent demands for fairness and transparency in AI-driven decision support systems.

In conclusion, while AI presents significant opportunities for enhancing healthcare, it also introduces complex ethical challenges that must be addressed. Privacy, consent, bias, transparency, and accountability are critical considerations that need to be carefully managed to ensure the responsible and equitable use of AI in healthcare.

### 7.2. Challenges in Integrating AI

The integration of AI in healthcare systems is not without its challenges. Among the most prominent are issues related to data security and interoperability. These challenges can impede the effective and safe use of AI in healthcare settings, and addressing them is crucial for the successful adoption of AI technologies. Some possible challenges in integrating AI include the following:

1. Data security concerns: As healthcare AI systems require access to large volumes of sensitive patient data, ensuring the security of these data is paramount [166]. The risk of data breaches and cyberattacks poses a significant concern. These security breaches can lead to the exposure of confidential patient information, resulting in privacy violations and potentially harming the trust between patients and healthcare providers. Implementing robust cybersecurity measures, including encryption, secure data storage solutions, and regular security audits, is crucial to protect patient data [167]. Additionally, educating healthcare staff about data security best practices is essential in safeguarding against breaches.
2. Interoperability between systems: Another major challenge in integrating AI into healthcare is the issue of interoperability—the ability of different healthcare IT systems and software applications to communicate, exchange data, and use the information that has been exchanged [168]. Many healthcare systems use a variety of electronic health record (EHR) systems and other digital tools that may not be compatible with one another or with new AI technologies. This lack of interoperability can hinder the seamless exchange of patient data, reducing the effectiveness of AI tools. Developing standardized data formats and communication protocols, as well as encouraging the adoption of interoperable systems, is vital to overcome this challenge [169].
3. Integration with existing clinical workflows: Integrating AI into existing clinical workflows can be challenging. Healthcare professionals may need to adjust their workflows to accommodate AI tools, which can be a time-consuming and complex process. Ensuring that AI systems are user-friendly and align with current clinical practices is essential to facilitate their adoption. Training and support for healthcare professionals in using these AI systems are also crucial for successful integration. For example, in a recent study, a three-tiered integration approach of AI-based image analysis into radiology workflows is outlined, focusing on enhancing automation and incorporating radiologist feedback for continuous AI improvement [170]. This approach entails initially visualizing AI outcomes without generating new patient records. It allows for the storage of AI-generated results in institutional systems and equips radiologists with tools to refine AI inferences for periodic retraining. This methodology was exemplified in a case study on brain metastases detection, where radiologist input substantially decreased false positives via iterative retraining with an expanded dataset.
4. Data quality and quantity: The effectiveness of AI systems depends heavily on the quality and quantity of the data they are trained on. Inconsistent, incomplete, or inaccurate data can lead to poor AI performance. Ensuring the collection of high-quality, comprehensive patient data is therefore a significant challenge in AI integration [171]. Standardizing data collection methods and ensuring thorough data curation processes are essential steps in addressing this issue.

### 7.3. Regulatory and Compliance Issues

The integration of AI into healthcare raises significant regulatory and compliance issues. Navigating this complex landscape is crucial for ensuring that AI applications in healthcare are safe, effective, and ethically sound. This subsection discusses the key regulatory and compliance challenges associated with AI in healthcare.

The regulatory framework for AI in healthcare is still evolving. Different countries and regions have varying standards and guidelines for the use of AI in medical settings [172,173]. For instance, in the United States, the Food and Drug Administration (FDA) is actively working on establishing clear guidelines for AI and machine learning-based medical devices [174]. Ensuring compliance with these regulations, which are often in a state of flux, is a challenge for AI developers and healthcare providers. Staying up to date with these developments and comprehending their relevance to AI applications is essential.

AI-based systems used in healthcare often require approval from regulatory bodies [175]. This process can be lengthy and complex, as it involves rigorous testing and validation of the AI models. Proving the safety and efficacy of AI systems to regulatory standards is a significant challenge, especially given the dynamic and evolving nature of AI algorithms. Regulatory bodies are increasingly focusing on the ethical implications of AI, including concerns about privacy, bias, and transparency. Ensuring that AI systems uphold these ethical standards and do not compromise patient safety is a key compliance issue.

Compliance with data protection and privacy laws is another major challenge. Laws such as the General Data Protection Regulation (GDPR) in the European Union and the Health Insurance Portability and Accountability Act (HIPAA) in the United States impose strict requirements on the handling of patient data [176]. AI systems that process patient data must comply with these laws, which involves implementing robust data protection measures and ensuring that patient data are used in a lawful and transparent manner.

Lastly, and critically, regulatory compliance for AI in healthcare extends beyond a mere initial approval. It demands continuous monitoring and reporting to ensure ongoing adherence to standards. This involves regular audits, necessary updates to AI algorithms to guarantee their correct functioning, and the immediate reporting of any adverse events or discrepancies to regulatory bodies.

## 8. The Future of AI in Healthcare

The rapid evolution of AI promises a transformative future for healthcare. This final section of this paper looks forward to the emerging trends and potential applications of AI in healthcare, examining how they might shape patient outcomes and the overall delivery of healthcare services. We will also explore the role of AI in responding to global health crises, such as pandemics, and its impact on public health strategies.

Table 5 thoroughly outlines the emerging trends and potential impacts of AI in healthcare. The subsequent sections further investigate and enhance understanding of these trends.

### 8.1. Personalized Healthcare Applications

Future research should continue to prioritize personalized healthcare applications. Possible future directions in this domain encompass the following:

1. Personalized medicine: One of the most promising trends in AI healthcare is the move towards more personalized medicine [177]. AI’s ability to analyze vast amounts of genetic, health data, and lifestyle information will enable the development of more precise and effective treatments tailored to individual patient profiles. This personalized approach can improve treatment outcomes and reduce side effects.
2. AI-powered tools for health and sleep monitoring: Future research should explore the development and validation of AI-driven tools and algorithms for the diagnosis, monitoring, and management of health issues and sleep disorders [178]. This includes leveraging machine learning to analyze data from wearable devices such as sleep patterns, heart rate variability, and activity levels. These analyses can, for example, help detect abnormalities such as sleep apnea and personalize treatment recommendations based on individual sleep profiles.
3. Longevity and aging: By harnessing the power of predictive analytics, AI can explore vast datasets to uncover biomarkers of aging and offer personalized strategies to slow or even reverse the aging process [179]. This includes leveraging AI for genomic interventions, where it could guide the editing of genes associated with aging mechanisms, enhancing cellular repair, resilience, and longevity. The potential of AI extends to the field of drug discovery and repurposing, where it can expedite the identification of compounds with anti-aging effects [180]. Moreover, AI’s integration into healthcare promises a paradigm shift towards preventive medicine, emphasizing early detection and intervention in age-related declines.

### 8.2. Enhanced Treatment Technologies

Future research should focus on AI-powered technologies for enhancing treatment methodologies. Some potential future directions include the following:

1. AI in drug discovery and development: AI is poised to play a significant role in accelerating drug discovery and development [181]. By rapidly analyzing molecular and clinical data, AI has the potential to identify potential drug candidates much faster than traditional methods. This acceleration could significantly reduce the time and cost associated with bringing new drugs to market.
2. Advanced robotics in surgery and rehabilitation: The use of AI-driven robotics in surgery and rehabilitation is expected to advance further [182]. Robotic systems, guided by AI algorithms, could potentially perform complex surgeries with high precision, reducing risks and improving patient outcomes. In rehabilitation, AI-powered exoskeletons and prosthetics are anticipated to offer greater mobility and independence to patients.
3. AI hardware accelerators: As AI applications in healthcare grow, the demand for efficient processing capabilities rises. AI hardware accelerators like GPUs, TPUs, and FPGAs optimize AI model performance, enabling real-time medical data processing with minimal latency. Integrating these accelerators into medical devices promises faster diagnosis, treatment planning, and analysis, thereby enhancing patient care outcomes. Developing dedicated AI hardware accelerators tailored to healthcare needs is a promising future direction for improving the efficiency and accessibility of AI-driven healthcare solutions.
4. AI-enhanced medical imaging: Future developments in AI are likely to produce even more advanced medical imaging techniques [183]. These advancements could provide clearer, more detailed images and enable the earlier detection of diseases, potentially even identifying health risks before symptoms appear.
5. Integrating AI with IoT and wearables: The integration of AI with the Internet of Things (IoT) and wearable technology is an emerging trend [184]. This combination could lead to real-time health monitoring systems that not only track health data but also provide proactive recommendations and alerts. AI can also be integrated into existing wearable technologies to provide further information regarding health and performance [185].

### 8.3. Healthcare System Optimization

In guiding future research, emphasis should be placed on healthcare system optimization, which can include the following:

1. Enhancing patient outcomes and system efficiency: The transformative potential of AI in healthcare can revolutionize patient care and system efficiency. Future AI applications aim to detect diseases earlier, customize treatments, and significantly personalize patient care, leading to improved recovery times and reduced mortality rates. AI’s role extends to optimizing healthcare resources, reducing costs, and improving care accessibility, especially for underserved communities [186]. Moreover, AI will support healthcare professionals by augmenting decision-making, promising equitable health improvements and a more efficient healthcare delivery system.
2. Global health monitoring systems: The significance of AI in addressing pandemics and global health emergencies is increasingly recognized as crucial [187]. By integrating and analyzing diverse data streams, AI is adept at quickly detecting the emergence of disease outbreaks, projecting their spread, and guiding effective public health interventions. During the COVID-19 pandemic, AI-powered models were used to predict the disease’s trajectory, showcasing the potential of AI in navigating the complexities of pandemic management [188]. Moreover, AI’s capabilities extend to enhancing public health strategies, enabling the expedited development and dissemination of vaccines and therapeutic solutions in times of crisis.

### 8.4. Data Management

Recognizing the critical role of data management, future research should prioritize its advancement. Data management involves the following:

1. Addressing data scarcity: The scarcity of labeled data in healthcare poses a significant challenge for AI development, especially in areas like rare disease research where data are inherently limited. A practical solution to this problem is the implementation of semi-supervised and weakly supervised learning techniques [189]. By utilizing a combination of a small set of labeled data and a larger volume of unlabeled data, these methods improve AI’s learning efficiency from minimal information, offering a viable strategy for advancing research and treatment in fields where comprehensive labeled datasets are scarce. However, for certain applications in healthcare, even obtaining a small amount of labeled data can be difficult. In such cases, emerging techniques in the field of machine learning offer intriguing possibilities.
  Few-shot learning: Few-shot learning requires only a small number of labeled examples for a new concept. This could be beneficial for situations where obtaining even a small amount of labeled data for a rare disease is possible. By learning from these few examples, the model could potentially generalize to similar cases [190,191].
  Zero-shot learning (ZSL): In theory, ZSL could allow AI models to learn about new diseases or medical conditions even with no labeled data for those specific cases. ZSL leverages existing knowledge and relationships between concepts to make predictions for unseen categories. While ZSL is still under development, it holds promise for healthcare applications where data are extremely limited [192].
  Meta-learning: This approach focuses on training models to “learn how to learn” efficiently. A meta-learning model could be trained on various healthcare-related tasks with limited datasets for each task. This acquired knowledge about learning itself could then be applied to new, unseen medical problems with minimal data, potentially improving performance [193].
2. Ensuring model versatility: Achieving versatility in AI models is essential for their effective application across the diverse landscape of healthcare settings and patient demographics. Techniques such as domain adaptation and transfer learning stand out as effective solutions, enabling AI models trained on one dataset to adjust and perform accurately on another with little need for retraining [194]. This capability is particularly valuable in healthcare, where patient characteristics, disease profiles, and treatment responses can vary widely [195]. By fostering such adaptability, these techniques ensure that AI can be deployed more universally, enhancing its effectiveness and utility for a broad spectrum of patients.

### 8.5. Ethical Considerations and Trust Building

Acknowledging the importance of ethical considerations and trust-building, future research should concentrate on these aspects. Ethical considerations and trust-building involve the following:

1. Ensuring data privacy: Addressing data privacy concerns in healthcare has become increasingly crucial with the rise in AI applications. An exemplary solution to this challenge is federated learning, a novel AI model training approach that enables algorithms to learn from data stored on local servers across different healthcare institutions without the need for direct data sharing [196]. This method significantly enhances privacy and security and offers a strategic advantage in the healthcare industry where the sensitivity and confidentiality of patient data are of utmost importance.
2. Stakeholder acceptance: Ensuring trust and acceptance among stakeholders is critical for the successful integration of AI into healthcare practices [197]. This encompasses not only patients and clinicians but also policymakers, regulatory bodies, healthcare administrators, and other relevant parties. Patients may express concerns regarding the reliability and accountability of AI-driven decision-making processes. Therefore, transparent communication about the role of AI in treatment plans and the potential benefits it offers is essential to foster patient acceptance. Similarly, clinicians may have reservations about entrusting AI algorithms with decision-making responsibilities, fearing loss of autonomy or professional judgment, as well as doubting the accuracy of AI decisions. Establishing comprehensive training programs and collaborative frameworks that empower clinicians to understand and validate AI tools effectively can mitigate these concerns. Furthermore, building trust extends to engaging stakeholders such as policymakers, regulatory bodies, and healthcare administrators. Transparency in AI development and deployment, coupled with clear communication about ethical, legal, and regulatory considerations, is crucial for gaining stakeholder trust. Establishing robust governance frameworks that address these concerns can enhance confidence in AI systems and ensure accountability.
3. Building trust with Explainable AI: Explainable AI (XAI) aims to make AI decision-making processes transparent and understandable to humans, a crucial aspect for clinical applications [198]. By providing insights into how AI models arrive at their conclusions, XAI fosters trust among healthcare professionals and patients, ensuring that AI-supported decisions are well informed and ethically sound. This transparency is vital for integrating AI into sensitive healthcare decisions, where understanding the rationale behind AI recommendations can significantly impact patient care and outcomes.

To sum up, the future of AI in healthcare is bright and filled with possibilities. While challenges remain, particularly in terms of ethics, regulation, and integration, the potential benefits are immense. As AI technology continues to evolve, it promises to revolutionize healthcare, making it more personalized, efficient, and responsive to global health needs.

## 9. Conclusions

This paper has provided an in-depth examination of the significant role played by AI in revolutionizing healthcare. Across various domains, including clinical decision-making, hospital operations, medical imaging, diagnostics, and patient care through wearable technologies and virtual assistants, AI has showcased its transformative impact. By enabling enhanced diagnostic accuracy, facilitating personalized treatments, and optimizing operational efficiency, AI holds promise for reshaping the healthcare landscape.

However, alongside these advancements, AI implementation in healthcare also raises important ethical considerations. Concerns surrounding data privacy, consent, and bias necessitate careful integration and adherence to regulatory standards. Balancing the potential benefits of AI with ethical considerations is imperative for ensuring its responsible and effective utilization in healthcare settings. In addition, equitable access and affordability are key building blocks for the future.

Looking towards the future, AI holds immense potential for personalized medicine, advanced drug discovery, and addressing global health crises. By leveraging AI technologies, healthcare delivery can become more efficient, data-driven, and patient-centric. Yet, realizing this potential requires a concerted effort from various stakeholders including technology developers, healthcare providers, policymakers, and patients.

## Figures and Tables

**Table 1:** Overview of advanced deep learning models in healthcare diagnosis and prognosis.

Algorithm Type	General Application	Limitations	Comments	Example
Convolutional Neural Networks (CNNs)	Image recognition and analysis in medical imaging (e.g., X-rays, MRI, CT scans)	Require large labeled datasets and substantial computational resources; can be a “black box” making interpretability difficult	Highly effective for spatial data; state of the art in medical image analysis	Deeplab v3+, a CNN variant for gastric cancer segmentation [28]. Results: 95.76% accuracy, outperforming SegNet/ICNet.
Recurrent Neural Networks (RNNs) and Long Short-Term Memory (LSTM) Networks	Analysis of sequential data such as ECG, EEG signals, or patient health records	Prone to overfitting on smaller datasets; long training times; difficulty in parallelizing the tasks	Suited for time-series data; LSTM addresses vanishing gradient problem in RNNs	LSTM for EEG signal classification [29]. Results: 71.3% accuracy, utilizing novel one-dimensional gradient descent activation functions for enhanced performance.
Transformer Models (e.g., BERT, GPT)	Natural language processing tasks, including clinical text analysis and patient history summarization	Require significant computational power and memory; pre-training on large datasets is time-consuming	Offer state-of-the-art performance in NLP; enable understanding of context in clinical documentation	Clinical-specific BERT (Transformer) for Japanese text analysis [30]: pre-trained on 120 million texts, achieving 0.773 Masked-LM and 0.975 Next Sentence Prediction accuracy, indicating potential for complex medical NLP tasks.
Generative Adversarial Networks (GANs)	Synthetic data generation for training models without compromising patient privacy; augmenting datasets	Training stability issues; generating high-quality data is challenging	Useful in data-limited scenarios; potential in creating realistic medical images for training	Differentially private GAN for synthetic data generation: utilizes convolutional AEs and GANs to produce realistic synthetic medical data, preserving data characteristics and outperforming existing models [31].
Graph Neural Networks (GNNs)	Modeling complex relationships and interactions between health data points (e.g., drug interaction prediction, disease progression modeling)	Complex model architectures that are difficult to interpret; scalability to very large graphs	Effective for data represented as graphs; emerging applications in personalized medicine	Knowledge-GNN for drug–drug interaction prediction: leverages knowledge graphs to capture complex drug relationships and neighborhood information, outperforming conventional models [32].

**Table 2:** Transformative applications of AI in hospital management.

Aspect	Applications
AI for hospital logistics and resource management	Predictive inventory management for medical supplies, medications, and equipment; efficient facility management including HVAC systems and predictive maintenance; optimization of resource allocation for staff and materials; and supply chain optimization and management during emergencies and health crises.
Automating administrative tasks with AI	Patient data management including EMRs and unstructured data analysis; billing and claims processing automation for accuracy and compliance; AI-driven scheduling systems for appointments and procedures; document management and processing automation; automated communication and reminders for patient engagement; and data security and compliance monitoring.
AI in patient flow and scheduling optimization	Optimization of patient flow through predictive analysis of admissions, discharges, and transfers; dynamic scheduling systems for appointments and procedures, minimizing no-shows and cancellations; reduction in waiting times through better triage processes and real-time patient wait time prediction; and enhancement of patient experience by providing accurate information and integrating with telehealth services for virtual consultations.

**Table 3:** Overview of AI applications in medical imaging.

Imaging Modality	Application	Example of AI System	Impact
MRI	AI applications in MRI analysis encompass detection of brain abnormalities, tumors, strokes, neurodegenerative diseases, and more. AI can analyze images and quantify the volume of affected areas.	An AI system analyzes MRI images to detect brain abnormalities, such as tumors or strokes, and quantifies their volume, aiding in treatment planning [118].	Improved detection of tumors, strokes, and neurodegenerative diseases; quantification of affected areas aids in treatment planning and disease monitoring.
CT	AI in CT scan interpretation includes detecting lung nodules, identifying fractures and hemorrhages, assessing stroke severity, and characterizing tumor progression. AI systems can process CT scans rapidly and accurately, aiding in timely diagnosis.	An AI model diagnoses lung cancer with high accuracy and reduced false positives, improving diagnostic precision [119].	Faster detection of life-threatening conditions; enhanced accuracy compared to traditional methods; potential to save lives in emergency situations.
X-ray	AI applications in X-ray enhance image analysis for tumor detection, improving accuracy and reducing false positives and negatives. AI systems serve as a second reviewer, enhancing the sensitivity of cancer screening.	AI-based CAD algorithms significantly improve radiologists’ sensitivity in breast cancer detection, reducing false negatives and improving cancer detection rates [120].	Increased sensitivity in detecting breast cancer lesions; reduction in false positives and negatives; enhancement of radiologists’ diagnostic accuracy.
Ultrasound	AI aids in analyzing echocardiography scans to assess cardiovascular function and detect structural abnormalities of the heart. AI systems measure parameters such as ejection fraction and aid in diagnosing and managing heart diseases.	A novel AI algorithm accurately calculates left ventricular ejection time in echocardiography, providing reliable metrics for cardiac function assessment [121].	Accurate assessment of cardiovascular parameters; reduction in user-dependent variability; enhancement of clinical utility in echocardiography.

**Table 4:** AI-powered technologies for patient care and monitoring.

Main Applications	Key Technologies and Applications	Benefits	Challenges
AI-powered wearable devices	Continuous physiological monitoring (heart rate, blood pressure, etc.); early detection of health issues; personalized recommendations for lifestyle changes	Improved patient engagement; proactive health management	Data collection and model deployment; balancing accuracy with wearable device limitations
Virtual nursing assistants	24/7 patient support and health reminders; chronic disease management; patient education and behavior monitoring	Enhanced patient engagement and education; improved treatment plan compliance	Data privacy and information accuracy; ensuring they complement human care
AI in telemedicine and remote patient engagement	Advanced diagnostics and consultations; personalized virtual consultations; remote patient monitoring and predictive analytics	Increased healthcare accessibility; proactive chronic condition care	Data privacy, system accuracy, and integration

**Table 5:** Emerging trends and potential impacts of AI in healthcare.

Trend/Application	Potential Impact	Challenges	Future Directions
Personalized medicine	Revolutionizes treatment for diseases with genetic components, significantly improving patient outcomes through customized care plans.	Data privacy, integration into clinical practice, and ensuring equitable access across diverse patient populations.	Expanding personalized medicine to encompass mental health, lifestyle diseases, and integrating real-time health monitoring data for dynamic treatment adjustments.
AI-powered tools for health and sleep monitoring	Improved detection and diagnosis of sleep disorders, early identification of potential health issues, personalized treatment, and proactive interventions.	Data privacy, accuracy of predictions, and user acceptance and comfort with interventional technologies.	Designing analysis and intervention technologies to monitor, predict, and manage health issues and sleep disorders; integration with wearable devices and smart home technology, providing real-time adjustments.
Longevity and aging	Unlocks new possibilities in aging research, promoting healthier, extended lifespans through AI-driven genomic interventions and predictive analytics for preventive medicine.	Addressing ethical implications of longevity research, ensuring accessibility and fairness in anti-aging treatments.	Leveraging AI for comprehensive health longevity platforms, integrating AI with regenerative medicine, and creating personalized anti-aging treatment plans based on predictive health analytics.
AI in drug discovery and development	Reduces time and costs in drug market introduction, enhances the efficacy of new drugs by identifying optimal candidate molecules.	Ensuring the reliability of AI predictions; ethical concerns around automated decision-making in drug development.	Leveraging AI to explore novel drug pathways, improve clinical trial design, and predict patient responses to treatments more accurately.
Advanced robotics in surgery and rehabilitation	Improves precision in surgeries and patient outcomes in rehabilitation, potentially reducing recovery times and healthcare costs.	Ethical considerations around autonomy; the need for robust training programs for medical staff on robotic systems.	Developing autonomous surgical robots, enhancing robotic systems with sensory feedback for improved rehabilitation outcomes, and expanding applications in minimally invasive procedures.
AI hardware accelerators	Faster diagnoses, treatment planning, and analysis, improved patient care outcomes, and real-time medical data processing.	Integration with medical devices; cost and power consumption of accelerators.	Develop healthcare-specific AI hardware; improve accessibility of AI-driven healthcare.
AI-enhanced medical imaging	Enables earlier and more accurate disease detection, potentially even identifying health risks before symptoms appear, thus shifting towards preventive healthcare models.	Balancing the need for patient privacy with the benefits of data sharing for AI training; integrating AI tools with existing healthcare infrastructures.	Developing AI systems capable of cross-modality analysis, improving 3D imaging techniques, and creating predictive models for disease progression based on imaging data.
Integrating AI with IoT and wearables	Leads to proactive health management and personalized health recommendations, potentially reducing emergency healthcare interventions.	Addressing data security and ensuring device interoperability across different healthcare systems.	Enhancing predictive analytics for early detection of health anomalies, creating an ecosystem of interconnected devices for holistic health monitoring; unobtrusive health monitoring.
Enhancing patient outcomes and system efficiency	Promises significant improvements in patient care through earlier disease detection, customized treatments, and optimized healthcare resource management.	Ensuring equitable improvements across all populations, addressing the digital divide in healthcare access.	Implementing AI-driven health advisories in public health strategies, optimizing healthcare delivery models with predictive resource allocation, and enhancing remote patient monitoring systems.
Global health monitoring systems	Strengthens global health security by enabling rapid response to disease outbreaks and guiding public health interventions with data-driven insights.	Integrating diverse data streams in real time, adapting models quickly to emerging health threats.	Developing global AI-powered surveillance systems, enhancing predictive models for epidemic and pandemic forecasting, and creating AI-driven platforms for vaccine and therapeutic development.
Addressing data scarcity	Facilitates AI development in under-researched areas, such as rare diseases, by making efficient use of limited data resources.	Creating effective models with sparse data, ensuring the generalizability of findings from limited datasets.	Exploring novel data augmentation techniques, crowdsourcing for data collection, and cross-institutional data sharing initiatives to enrich datasets. Developing advanced techniques based on few-shot leaning.
Ensuring model versatility	Allows for the broader application of AI models across varying healthcare settings and patient demographics, improving the universality and accessibility of AI-driven healthcare solutions.	Developing adaptable models that maintain high accuracy across diverse datasets, addressing potential biases in AI training.	Advancing transfer learning and domain adaptation techniques that can be personalized at the point of care.
Ensuring data privacy	Enhances privacy and security in healthcare applications, addressing one of the major concerns of digital health data management.	Balancing the utility of data for AI training with stringent privacy requirements, adapting regulations to keep pace with technological advancements.	Developing more advanced privacy-preserving AI techniques, such as secure multi-party computation, federated learning, and advanced encryption methods for health data.
Stakeholder acceptance	Successful AI integration in healthcare; improved trust and collaboration.	Concerns about AI reliability and clinician autonomy.	Transparent communication and training programs.
Building trust with Explainable AI (XAI)	Enhances the trustworthiness of AI systems among healthcare professionals and patients, ensuring that AI-supported decisions are well informed and ethically sound.	Simplifying complex AI decision-making processes for non-technical stakeholders, ensuring explanations are meaningful and actionable.	Integrating XAI into clinical workflows, developing standards for AI explanations in healthcare, and educating healthcare professionals on interpreting AI decisions.

## Data Availability

Not Applicable.
